# The influence of brachytherapy on tooth development: a longitudinal study of pediatric head and neck tumor survivors

**DOI:** 10.1186/s12903-026-07804-x

**Published:** 2026-03-20

**Authors:** Guohao Zhang, Wenting Yu, Huifang Yang, Guanxi Wu, Zezhao Liu, Liang Wang, Xiangqin Liu, Yuxing Bai, Jianguo Zhang, Lei Zheng, Chuanbin Guo, Mingwei Huang

**Affiliations:** 1 Department of Oral and Maxillofacial Surgery, Peking University School and Hospital of Stomatology & National Center for Stomatology & National Clinical Research Center for Oral Diseases & National Engineering Research Center of Oral Biomaterials and Digital Medical Devices, No.22, Zhongguancun South Avenue, Haidian District, Beijing, 100081 PR China; 2 Department of Orthodontics, Beijing Stomatological Hospital, School of Stomatology, Capital Medical University, Beijing, 100070 PR China; 3 Center of Digital Dentistry, Peking University School and Hospital of Stomatology & National Center for Stomatology & National Clinical Research Center for Oral Diseases & National Engineering Research Center of Oral Biomaterials and Digital Medical Devices, Beijing, 100081 PR China; 4 Department of Stomatology, Aerospace Center Hospital, Beijing, 100049 PR China; 5 Department of Oral and Maxillofacial Surgery, The No.2 Hospital of Baoding, Baoding, Hebei 071051 PR China; 6 Faculty of Dentistry, National University of Singapore, Singapore, 119085 Singapore

**Keywords:** Tooth development, Brachytherapy, Head and neck tumor, Developmental anomalies, Pediatric and adolescent tumor, Longitudinal study

## Abstract

**Background:**

Interstitial brachytherapy is an effective and organ-preserving radiotherapeutic modality for pediatric and adolescent patients with head and neck tumors, offering favorable outcomes with minimal impact on surrounding normal tissues. However, the long-term effects of brachytherapy on dental development in young patients remain underexplored, despite clinical observations of tooth anomalies in tumor survivors. This study aimed to provide longitudinal outcomes of tooth developmental anomalies in pediatric and adolescent tumor survivors who underwent head and neck interstitial brachytherapy and to investigate the relevant factors.

**Methods:**

The longitudinal panoramic radiographs of the patients at different ages before brachytherapy and during follow-up were evaluated. A modified Demirjian staging technique was used to allocate a developmental stage for each tooth, and the defect index classification criteria were applied to describe the severity of tooth damage. Logistic regression analysis was performed to identify factors associated with long-term dental abnormalities.

**Results:**

A total of 210 developing permanent teeth from seven patients were included and evaluated. There were 31 teeth (14.76%) that exhibited developmental anomalies. The incidence and severity of tooth developmental abnormalities after brachytherapy were jointly determined by the radiation dose received by a given tooth and its developmental stage at the time of treatment. Multivariable analysis indicated that D_2cc_ had the largest effect size (OR 1.029, 95% CI 1.017–1.042) and the most significant statistical association (*P* = 0.002). When D_2cc_ was 0–10 Gy, the incidence of tooth developmental abnormalities was 1.90%; and when D_2cc_>100 Gy, the incidence reached 85.7%. The maxillary region with implanted radioactive seeds had a significantly higher risk of dental abnormalities compared to other regions.

**Conclusion:**

This study suggests that interstitial brachytherapy may contribute to dental developmental alterations in pediatric and adolescent patients, with severity potentially influenced by radiation dose and tooth developmental stage. D_2cc_ appeared to be the most informative dose-volume parameter, with lower risks observed at < 10 Gy and higher risks at > 100 Gy. Although limited by sample size and retrospective design, these findings underscore the importance of considering developing dentition when planning individualized radiotherapy for young patients.

**Supplementary Information:**

The online version contains supplementary material available at 10.1186/s12903-026-07804-x.

## Background

Brachytherapy, which has been shown to be safe and effective for treating sarcoma, desmoid tumor, and salivary gland tumor, is crucial in the treatment of head and neck tumors in children and adolescents [1–5]. Interstitial brachytherapy offers target conformality and minimally invasive strategies, resulting in favorable tumor treatment outcomes while minimizing side effects on surrounding normal tissues of head and neck, thereby maximizing the preservation of organs and tissues in pediatric and adolescent patients.

Radiotherapy to the head and neck of children and adolescent patients can result in severe adverse effects, including jaw radionecrosis, malocclusion, radiogenic caries, xerostomia, craniofacial growth and development impairment, and facial asymmetrical deformities [6–8]. The quality of life for survivors of pediatric and adolescent tumors largely depends on the anatomical integrity and physiological functionality of the oral and maxillofacial region following treatment.

The presence of dental anomalies can impair speaking, chewing, and facial aesthetics in childhood and teenage patients, resulting in a lower standard of life. Prior research indicates that head and neck radiotherapy in conjunction with chemotherapy elevates the prevalence of dental anomalies in young patients [9–11]. Longitudinal research on the long-term impact of brachytherapy on tooth development in children and teenage patients is still lacking. Clinical follow-up examinations indicate that tooth developmental problems have arisen in certain tumor survivors who underwent brachytherapy. This study aimed to investigate the long-term longitudinal outcomes of tooth developmental anomalies in pediatric and adolescent tumor survivors who underwent head and neck interstitial brachytherapy and to investigate the relevant factors. The longitudinal panoramic radiographs were assessed to examine the developmental abnormalities of permanent teeth.

## Methods

### Study sample

The study group included pediatric and adolescent patients with head and neck tumors who underwent brachytherapy at Peking University School and Hospital of Stomatology from September 2014 to September 2017. The criteria for inclusion and exclusion were as follows: patients who received brachytherapy before the age of 18 and those for whom at least two panoramic radiographs were available, with a minimum interval of one year between the first and last imaging were included; patients with systemic diseases that could potentially affect growth and development, as well as those with a history of chemotherapy, external beam radiotherapy or orthodontic treatment were excluded. In total, seven patients were included, and their information was shown in Table 1.

**Table 1:** Key characteristics of the patients

No.	Sex	Histology	Tumor localization	Treatment modality	Age at BT (years)	Panoramic number		Panoramic age (years)
1	male	sialoblastoma	maxilla	BT	0.9		3	7.0–10.8
2	male	desmoid tumor	mandible	SG + BT	2.8		7	2.4–9.0
3	female	desmoid tumor	temporomandibular region	SG + BT	3.3		2	3.4–7.7
4	female	desmoid tumor	temporomandibular region	BT	3.3		4	3.8–11.1
5	male	acinic cell carcinoma	salivary gland	SG + BT	4.0		3	5.6–10.1
6	male	malignant fibrous histocytoma	maxilla	SG + BT	5.8		3	8.8–16.6
7	male	malignant solitary fibrous tumor	mandible	SG + BT	9.3		4	9.3–10.8

*BT* brachytherapy, *SG* surgery

### Treatment modality

The patients received interstitial LDR brachytherapy for head and neck tumors between 0.9 and 9.3 years of age (11–112 months), with treatment modalities including surgery combined with brachytherapy or brachytherapy alone. For patients treated with surgery combined with brachytherapy, surgical resection was performed first (Table S1), followed by interstitial brachytherapy.

Permanent implantation of ^125^I seeds was carried out using implantation needles inserted directly into the tumor bed. The implanted ^125^I seeds (type 6711; Beijing Atom and High Technique Industries, Beijing, China) had an activity of 0.5–0.7 mCi, a half-life of 59.4 days, and delivered effective radioactivity for approximately six months. Following acquisition of CT imaging data, treatment planning was performed using a brachytherapy treatment planning system (BTPS; Beijing Astro Technology Ltd. Co., Beijing, China). After importing the imaging data, the gross tumor volume (GTV) was delineated, and the planned target volume (PTV) was defined to include the GTV and the surrounding subclinical disease. Once the target was determined, the positions of the trajectories of the implantation needles and the radioactive seeds were planned to avoid major vessels, critical tissues, and organs, while achieving a highly conformal and homogeneous dose distribution that minimized exposure to adjacent normal tissues. The implantation was performed using a spatial configuration of 1.0–1.5 cm between planes and 1.0–1.5 cm between channels. During the procedure, implantation needles were inserted along the preplanned pathways under guidance from individualized templates or navigation systems, and the radioactive seeds were delivered accordingly. A postoperative CT scan was obtained to verify seed positions and perform dosimetric verification [12, 13].

The prescription dose for brachytherapy ranged from 110 to 140 Gy. Dosimetric evaluation parameters included D_90_ (the minimum dose received by 90% of the PTV), V_100_ (the percentage of the PTV receiving 100% of the prescription dose), and V_150_ (the percentage of the PTV receiving 150% of the prescription dose), as summarized in Table S2. After receiving brachytherapy, patients underwent regular follow-up evaluations. Clinical and imaging examinations were performed to assess the tumor response to brachytherapy and to monitor potential adverse effects. If any suspicious mass was detected on clinical or imaging assessment, surgical biopsy or needle biopsy was conducted to determine whether tumor recurrence had occurred. In accordance with the Common Terminology Criteria for Adverse Events (CTCAE) version 6.0, we conducted a comprehensive assessment of adverse events in head and neck region.

### Radiographic analysis

We retrospectively collected 2 to 7 panoramic radiographs for each patient, taken at different ages before brachytherapy and during follow-up. Before analyzing the imaging data, all radiographic files were anonymized, and patient names were replaced with study-specific codes to ensure full protection of patient privacy. A tooth-by-tooth evaluation of permanent teeth was conducted in the panoramic radiographs. Teeth with buccolingual inclination that hindered clear observation of root development were excluded from the analysis.

**Fig. 1:**
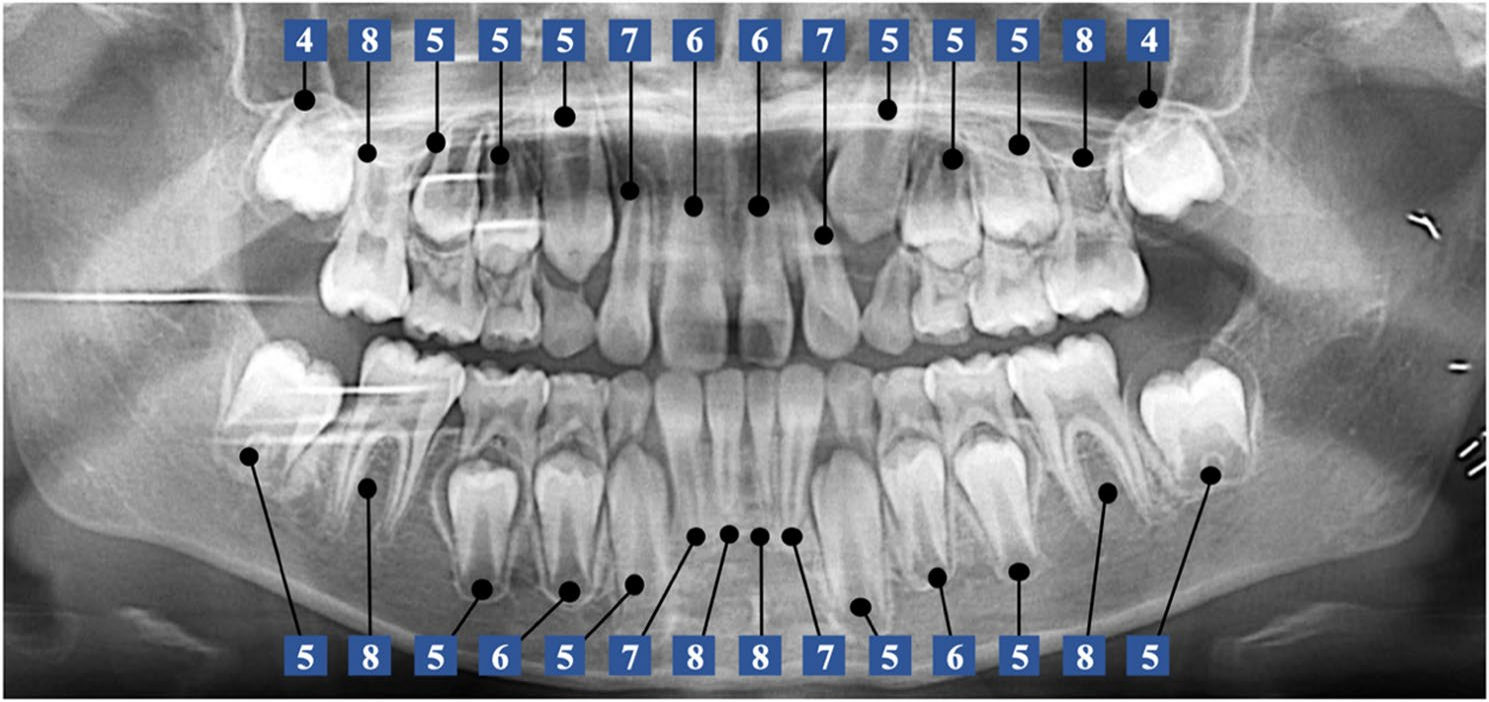
A modified Demirjian staging technique was used to allocate a developmental stage for each tooth

The FDI numbering system was applied for teeth numbering [14]. The longitudinal panoramic radiographs were evaluated, a modified Demirjian staging technique was used to allocate a developmental stage for each tooth [15] (Fig. 1). The development of permanent teeth was categorized into ten stages (Table 2). All radiographs were separately evaluated by two experts possessing substantial clinical experience and training. Imaging data within two months after brachytherapy, which were used to verify radiation dose distribution, were applied to assess the developmental stage of the teeth at the initiation of brachytherapy.

**Table 2:** Modified Demirjian descriptive allocation criteria for developmental stages in teeth

Stage	Single-rooted teeth	Multi-rooted teeth
**0**	The developmental crypt is visible in the jawbone. Calcification has yet to begin	The developmental crypt is visible in the jawbone. Calcification has yet to begin
**1**	Start of calcification is visible at the superior level of the crypt in the form of a cone	Start of calcification is visible at the superior level of the crypt in the form of small cones. The calcified points are not fused together
**2**	The calcified point forms one uniform cusp presenting a regularly outlined incisal edge	The calcified points are fused and present a regularly outlined occlusal surface
**3**	a Incisal enamel formation is complete. Extension towards the cervical region is seen	a Occlusal enamel formation is complete. Extension towards the cervical region is seen
	b Dentinal deposition has started	b Dentinal deposition has started
	c The coronal outline of the pulp chamber can be seen as a radiolucent thick line in the center of the crown	c The pulp chamber can be seen as a radiolucent curved outline at the occlusal border
**4**	a Crown formation is completed down to the cemento-enamel junction	a Crown formation is completed down to the cemento-enamel junction
	b Pulp chamber outline has a thick rectilinear shape	b Pulp chamber outline has a trapezoidal shape. The projections of the pulp horns create an umbrella like shape
**5**	a Beginning of root formation is seen as an extension of dentine and cementum deposit downwards from the cemento-enamel junction	a Beginning of root formation is seen as a spicule
	b The root length is less than the crown height	b The root length is less than the crown height
		c A calcified point or semi-lunar shape shows initial formation of the root bifurcation
**6**	Root length is equal to or greater than the crown height	a Roots are more defined with funnel shaped endings
		b Root length is equal to or greater than the crown height
**7**	Walls of the root canal are parallel; the apical end is still partially open	Walls of the root canal are parallel; the apical end is still partially open
**8**	Walls of the root canal are converging at the apex; the apical end is still partially open	Walls of the root canal are converging at the apex; the apical end is still partially open
**9**	a The apical end of the root canal is completely closed	a The apical end of the root canal is completely closed
	b The periodontal ligament has a uniform width around the entire root	b The periodontal ligament has a uniform width around the entire root

The panoramic radiograph taken at the last follow-up was used to evaluate the occurrence of dental abnormalities. The defect index (DeI) classification criteria were modified and applied to describe the severity of damage to the tooth [16]. Each tooth was categorized using the criteria in Table 3. The severity of dental abnormalities increases with the grading scale.

**Table 3:** Criteria of dental abnormalities

Class	Allocation criteria
ND	not determined
	(a) developing teeth with an unclear final outcome; teeth with arrested development are not classified as ND
	(b) missing teeth not categorized in the aplasia group because of young age
	(c) teeth not reliably seen on radiograph
D0	R/C ratio > 1.6; no disturbance
D1	R/C ratio 1.2–1.6; mild disturbance
D2	R/C ratio 0.9–1.1; severe disturbance
D3	R/C ratio < 0.9; very severe disturbance
D4	microdontia; exceptionally small tooth
D5	aplasia; missing tooth
	a tooth was not considered missing before the following ages:
	first premolar: <5 years; second premolar: <6 years; second molar: <6 years; third molar: <13 years

R/C ratio: the ratio of the length of tooth root to tooth crown

We evaluated the consistency between the two experts’ radiographic assessments. For teeth with inconsistent grading, the two experts conducted focused discussions and, with reference to both the pictorial atlas and the written criteria, jointly determined the most accurate grade.

### Dose distribution of teeth

**Fig. 2:**
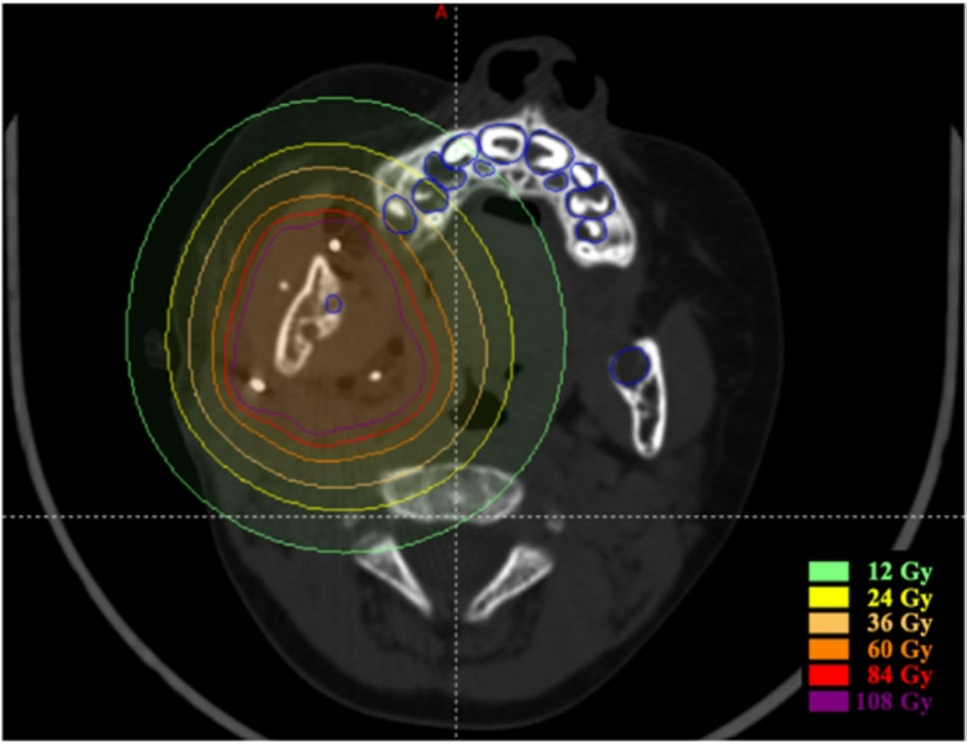
The radiation dose received by each permanent tooth was calculated in the BTPS

The radiation dose received by each permanent tooth was calculated using the BTPS (Fig. 2). CT data acquired within two months after brachytherapy were imported into the BTPS, and the region of each permanent tooth was manually contoured to calculate D_mean_ (the average radiation dose received by the tooth), D_max_ (the highest radiation dose received by any part of the tooth), and D_2cc_ (the mean radiation dose received by the 2 cc of tooth with the highest exposure).

### Statistical analysis

Logistic regression analysis was performed to identify factors associated with long-term dental abnormalities. Age at brachytherapy, sex, tumor site, treatment modality, D_2cc_, D_mean_, and D_max_ to the tooth region were included. Variables with *P* < 0.05 in univariable analyses were included in the multivariable analyses. Results were presented as odds ratios (OR) with 95% confidence interval (CI). Both univariable analysis and multivariable analysis could not be explored for the tumor site at the salivary gland due to no dental abnormality events in this subgroup. Statistical analysis was conducted using SPSS software (SPSS version 24.0) .

## Results

**Fig. 3:**
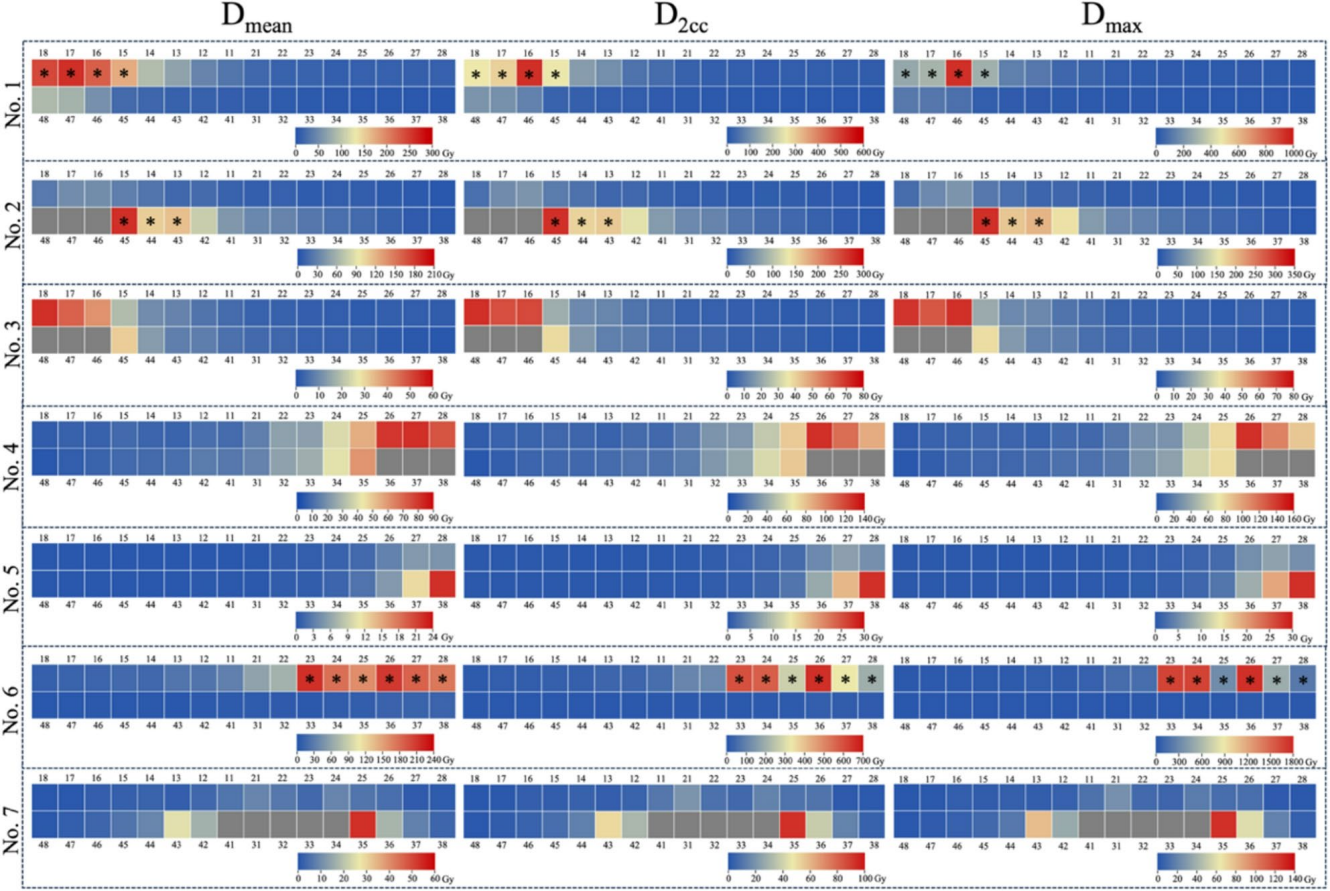
The heatmap illustrated the radiation dose received by each permanent tooth. The boxed panel showed the radiation doses delivered to all teeth of a representative patient. The horizontal labels at the top indicated the dosimetric parameters: Dmean, the average radiation dose received by the tooth; D2cc, the mean radiation dose received by the 2cc of tooth with the highest exposure; and Dmax, the highest radiation dose received by any part of the tooth. The tooth identifier corresponding to each heatmap cell was labeled above or below the cell. The numerical dose value represented by each cell color was indicated beneath the heatmap. Blue colors indicated the tooth reveived lower radiation doses, red colors indicated the tooth reveived higher doses, and grey color indicated teeth that were not included in the analysis. The teeth located within the target volume were marked with *

A total of 210 permanent teeth from seven patients were included and analyzed in the study. At the start of brachytherapy, the permanent teeth were at different developmental stages, as detailed in Table S3. Due to variations in the position, number, and radioactivity of the implanted radioactive seeds, the teeth received different levels of radiation dose during brachytherapy (Fig. 3). The D_mean_ ranged from 0.0 to 258.9 Gy, D_2cc_ ranged from 0.0 to 655.4 Gy, and D_max_ ranged from 0.0 to 1760.2 Gy. The Cohen’s kappa values for two experts’ radiographic assessments were: modified Demirjian staging technique: κ = 0.91 (95% CI: 0.87–0.95, *P* < 0.001) and DeI scoring criteria: κ = 0.93 (95% CI: 0.87–0.99, *P* < 0.001), indicating excellent interobserver reliability for both assessments.

Long-term follow-up after brachytherapy revealed that there were 31 teeth (14.76%) that exhibited developmental anomalies, and the distribution of dental abnormalities was shown in Table 4, which was categorized according to the panoramic radiograph taken at the last follow-up. When D_2cc_ was 0–10 Gy, the incidence of tooth developmental abnormalities was 1.90%; at 10–50 Gy, the incidence increased to 8.96%; at 50–100 Gy, it rose to 29.41%; and when D_2cc_ exceeded 100 Gy, the incidence reached 85.70%. The severity of dental developmental abnormalities increased with higher radiation dose received by the teeth. When exposed to similar level of radiation dose, teeth at earlier developmental stages tended to exhibit more severe developmental abnormalities.

**Table 4:** Distribution of dental abnormalities

D_2cc_	Dental age at BT	Dental abnormalities classification and the number of affected teeth						
		D0	D1	D2	D3	D4	D5	ND
0–10 Gy	NO	1					2	16
	0–7	13						73
10–50 Gy	NO					1	2	8
	0–1							7
	2		2					11
	3	1	1					14
	4–7	10						11
50–100 Gy	NO						3	2
	2			1				2
	3							3
	4		1					1
	5	1						2
> 100 Gy	NO					1	4	2
	0					1		
	1				1			
	2			1		2		
	3		1		1			
	4		1		5			
	5	1						

D_2cc_: the mean radiation dose received by the 2 cc of tooth with the highest exposure; *NO* teeth not reliably observed on radiograph, *BT* brachytherapy, *ND* not determined

Univariate logistic regression analysis indicated that several variables were significantly associated with long-term dental developmental abnormalities, including tumor site, treatment modality, and radiation dose (Table 5). Specifically, patients with tumors located in the maxilla had a significantly higher risk of post-treatment dental abnormalities compared to those with tumors in other regions (Mandible OR 0.163, 95% CI 0.052–0.512, Temporomandibular region OR 0.314, 95% CI 0.121–0.814). Notably, in the case where the tumor (and thus the site of seeds implantation) was located in the parotid gland, no dental developmental abnormality was observed. However, for the purpose of maintaining statistical power, the site was included in the univariate analysis. Compared with patients who received brachytherapy alone, those who underwent surgery combined with brachytherapy (OR 0.369, 95% CI 0.169–0.805) had a significantly lower risk of subsequent dental abnormalities, which may be attributed to the higher radiation doses typically delivered in brachytherapy-only regimens. D_2cc_ (OR 1.032, 95% CI 1.020–1.045), D_mean_ (OR 1.042, 95% CI 1.026–1.059) and D_max_ (OR 1.028, 95% CI 1.018–1.039) were found to be significant risk factors.

**Table 5:** Logistic regression analysis of factors associated with dental abnormalities

	Univariable analysis			Multivariable analysis		
	OR	95% CI	*P*-value	OR	95% CI	*P*-value
Age at brachytherapy (years)	0.827	0.684–1.000	0.050			
Sex						
Male	Ref.					
Female	0.343	0.114–1.028	0.056			
Tumor site						
Maxilla	Ref.			Ref.		
Mandible	0.163	0.052–0.512	0.002	0.972	0.195–4.842	0.972
Temporomandibular region	0.314	0.121–0.814	0.017	0.271	0.068–1.084	0.065
Salivary gland	/	/	/	/		
Treatment modality						
BT	Ref.			Ref.		
BT + SG	0.369	0.169–0.805	0.012	0.312	0.073–1.338	0.117
Tooth region, D_2cc_ (Gy)	1.032	1.020–1.045	< 0.001	1.029	1.017–1.042	< 0.001
Tooth region, D_mean_ (Gy)	1.042	1.026–1.059	< 0.001			
Tooth region, D_max_ (Gy)	1.028	1.018–1.039	< 0.001			

*OR* odds ratio, *CI* confidence interval, *BT* brachytherapy treatment, *SG* surgery treatment; D_2cc_: the radiation dose received by the 2 cc of tooth with the highest exposure; D_mean_: the average radiation dose received by the tooth; D_max_: the highest radiation dose received by any part of the tooth

In the multivariable analyses, D_2cc_ was included as surrogates for the other dosimetric parameter to avoid multicollinearity. This was based on the correlation matrix and Variance Inflation Factor (VIF) showing strong correlation between D_2cc_ to the D_mean_ and D_max_. Moreover, D_2cc_ is consistent with the “volume effect” principle in radiobiology and exhibited an even stronger independent association with dental developmental anomalies. The multivariable analyses identified D_2cc_ (OR 1.029, 95% CI 1.017–1.042) as significantly associated with long-term dental developmental abnormalities, while tumor location and treatment modality were not (Table 5). We plotted the receiver operating characteristic (ROC) curve (Fig. S1) for the multivariable regression model and evaluated its performance metrics. The results indicated that the constructed model demonstrated excellent discriminatory ability, with an AUC of 0.925 (95% CI: 0.872–0.979, *P* < 0.001), a sensitivity of 83.9%, a specificity of 95.0%, and a Youden index of 0.789. It is noteworthy that in the multivariable model, tumor site and treatment modality were no longer statistically significant mainly because they are patient-level variables, with only seven cases available for analysis, which limited statistical power. Moreover, their effects on dental abnormalities are largely mediated through dose distribution. Once D_2cc_ was included in the multivariable model, the effects of tumor site and treatment modality were largely accounted for by the dosimetric factor, and their independent associations were no longer statistically significant.

**Fig. 4:**
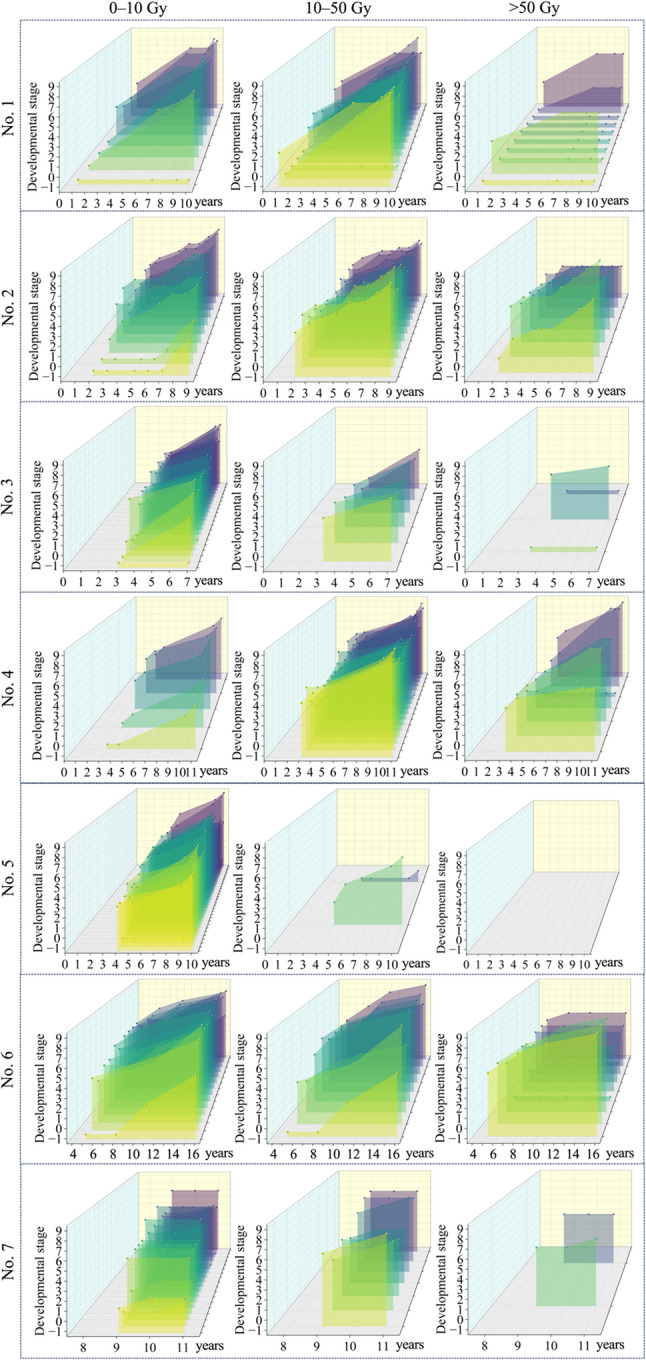
The trajectories of tooth development at different radiation dose levels. The boxed panel illustrated the developmental trajectories of teeth from a patient, categorized according to the D_2cc_ radiation dose received: 0–10 Gy, 10–50 Gy, and > 50 Gy. For each graph, the x-axis represented the patient’s age, and the y-axis represented the tooth developmental stage based on the modified Demirjian staging system. Teeth that were not reliably identified on radiographs were assigned a value of -1. The color gradient from yellow to purple represented a gradual increase in radiation dose within each graph

The post-brachytherapy developmental trajectories of teeth over time are illustrated in Fig. 4, with the longest follow-up period extending up to 10 years. As the radiation dose received by the teeth increased, the rate of tooth development slowed. Within the same dose range, younger dental age at the time of brachytherapy was more frequently associated with delayed or arrested tooth development during follow-up. When the radiation dose was below 10 Gy, virtually no adverse effects on tooth development were observed.

In accordance with the Common Terminology Criteria for Adverse Events (CTCAE) version 6.0, the evaluation results of dental adverse events were presented in Table S4. Moreover, no instances of other late adverse effects, such as soft-tissue necrosis, fibrosis, or osteoradionecrosis, were observed during follow-up.

## Discussion

In the treatment of pediatric and adolescent tumor patients, the primary goal is to ensure long-term survival while minimizing serious disruptions to the patient’s growth and development. Compared with conventional external beam radiotherapy, brachytherapy offers advantages such as high precision, target conformality, and improved local control rates [17–20]. However, the late effects of brachytherapy on tooth development remain unclear. Minimizing radiation exposure to healthy tissues surrounding the tumor and preserving oral function and aesthetics are critical to improving the long-term quality of life in pediatric cancer survivors [21]. Moreover, teeth in different positions exhibit distinct developmental rates and stages at the time of tumor treatment, which further complicates the quantitative analysis of how brachytherapy impacts tooth development in pediatric patients. Although studies have demonstrated that developing teeth are sensitive to radiation and that radiotherapy can lead to delayed or arrested dental development [9, 22, 23], few longitudinal studies have systematically tracked the growth patterns of the entire dentition and the associated influencing factors following brachytherapy.

As the study was a descriptive, exploratory observational study intended to document the real-world impact of a broad radiation dose spectrum on developing teeth in children with head and neck tumors, teeth located within the target volume were also included in the analysis. According to our long-term follow-up study, the majority of teeth did not exhibit severe developmental impairment after brachytherapy. Due to the steep dose fall-off characteristic of brachytherapy in regions surrounding the target area, even teeth located on the same side or within the same jaw may show markedly different developmental outcomes—ranging from normal growth to complete arrest of development. Among the tumor survivors in this study, a total of 31 teeth (14.76%) were observed to have developmental abnormalities. The most severe abnormality was tooth agenesis (D5). Other abnormalities included microdontia (D4), very severe developmental disturbances (D3, R/C ratio < 0.9), severe disturbances (D2, R/C ratio 0.9–1.1), and moderate disturbances (D1, R/C ratio 1.2–1.6). Notably, in addition to the classical DeI scoring criteria, this study also incorporated longitudinal Demirjian staging to enable precise classification of dental abnormalities. If a tooth showed no change in Demirjian stage across multiple longitudinal time points, it was defined as having growth arrest. Such teeth were scored as D0–D3 in the final panoramic radiograph at the last follow-up, rather than being classified as “not determined,” even if the Demirjian score had not reached stage 9.

The results of this study indicate that the severity of tooth developmental abnormalities after brachytherapy were jointly determined by the radiation dose received by a given tooth and its developmental stage at the time of treatment. Teeth in earlier stages of development are more susceptible to radiation-induced impairment, with even relatively low dose capable of disrupting teeth growth. Conversely, teeth that are near completion of development may continue to grow despite exposure to higher radiation dose. Notably, we observed that teeth exposed to moderate radiation dose exhibited slower development compared to their contralateral counterparts. However, this slower growth was not indefinite. Once the contralateral tooth ceased development, the delayed tooth also stopped growing. In addition, the relative position relationship between the tooth and the site of radioactive seeds, as well as the overall treatment strategy (brachytherapy alone or surgery combined with brachytherapy), may influence the extent of tooth developmental abnormalities.

The radiation dose was identified as the most critical determining factor. In univariate logistic regression analysis, all the three dose parameters showed a significant association between increasing radiation dose and the occurrence of tooth developmental abnormalities. Multivariate logistic regression further indicated that D_2cc_ had the largest effect size and the most statistically significant association, making it the most suitable parameter for identifying key risk factors. Using D_2cc_ as the dose parameter, we found that when the radiation dose was < 10 Gy, dental development was virtually unaffected. Among the 105 tooth positions analyzed within this dose range, only two teeth were missing—12 and 22 in the same patient, who was 9.3 years old at the time of brachytherapy. We hypothesize that these missing teeth were more likely due to congenital agenesis rather than radiation-induced loss. When the radiation dose ranged from 10 to 100 Gy, the incidence of dental abnormalities was 13.10%. However, when the dose exceeded 100 Gy, the incidence increased sharply to 85.7%. Therefore, a D_2cc_ of < 10 Gy is considered a safe radiation dose threshold that does not significantly impair tooth development, while a D_2cc_ >100 Gy indicates a high risk of dental developmental abnormalities. Understanding this threshold provides valuable guidance for clinicians in planning head and neck radiotherapy and in conducting long-term oral health follow-up for pediatric and adolescent patients.

In univariate logistic regression analysis, the location of radioactive seeds showed a significant association with the occurrence of tooth developmental abnormalities. Brachytherapy administered in the maxillary region was associated with a 6.1-fold higher risk of tooth abnormalities compared to the mandibular region, and a 3.2-fold higher risk compared to the temporomandibular region. This may be related to the richer blood supply in the maxilla. Radiotherapy exerts its effects by inducing DNA damage through ionizing radiation, a process that depends on the presence of oxygen [24]. Tissues with greater vascularization typically have higher oxygen levels, making radiation-induced DNA damage—and consequently, adverse effects on tooth development—more pronounced [25, 26]. Interestingly, in patients who received brachytherapy in the parotid region, no abnormalities in tooth development were observed. This could be attributed to the shielding effect of the jawbone, which may attenuate radiation exposure to adjacent dental structures.

In this study, the treatment regimens primarily included brachytherapy alone and brachytherapy combined with surgery. Univariate analysis revealed a higher risk of dental developmental abnormalities in the brachytherapy-alone group. However, this association was not observed in multivariate analysis. This discrepancy is likely due to the fact that patients treated with brachytherapy alone typically received higher radiation doses. Thus, the apparent difference in dental outcomes by treatment modality is likely a reflection of underlying differences in radiation exposure.

However, this study has several limitations. First, the sample size was small, with only seven eligible patients included, resulting in limited statistical power and restricting the generalizability of the findings. Second, as a retrospective study, the analysis was constrained by the information available in the medical records; several potentially influential factors—such as tumor size, anatomical location of the target volume, inter-individual variability in growth potential, and oral hygiene status—could not be adequately assessed. Third, manual contouring of tooth structures may introduce measurement variability despite careful review by experts. Fourth, the study lacked functional or clinical outcome correlations, such as mastication, occlusal function, or aesthetic assessments, which would provide important contextualization of the radiographic findings. In this study, we already included all eligible cases from our institutional database that met the predefined inclusion and exclusion criteria; due to the rarity of the diseases, challenges in long-term follow-up, and limited patient compliance, only seven longitudinal cases were available for analysis. To address these limitations, we have planned a prospective cohort study aimed at enrolling a larger patient population and systematically collecting comprehensive long-term follow-up data, including a broader range of potential influencing factors as well as detailed assessments of craniofacial aesthetics and oral-maxillofacial function. Artificial intelligence-based methods will also be applied for image segmentation and data analysis. We anticipate that such a study will enable more statistically robust and clinically informative fingings.

## Conclusion

This longitudinal study suggested that interstitial brachytherapy may be associated with varying degrees of dental developmental alterations in pediatric and adolescent patients. The observed severity appeared to correlate with both the delivered radiation dose and the developmental stage of the tooth at the time of treatment. Among the evaluated dose-volume parameters, D_2cc_ seemed to be the most informative indicator for identifying potential risk factors. In this cohort, a D_2cc_ of < 10 Gy might represent a dose range with relatively low risk of appreciable dental developmental disturbances, whereas a D_2cc_ >100 Gy was associated with a higher likelihood of dental abnormalities. These findings should be interpreted cautiously given the limited sample size and retrospective nature of the study, but they highlight a potential need for individualized radiotherapy planning that considers the vulnerability of developing dentition to better support long-term oral health in young cancer survivors.

## Supplementary Information

Supplementary Material 1

## Data Availability

The datasets used and/or analysed during the current study are available from the corresponding author on reasonable request.

## Abbreviations

BTPS: Brachytherapy treatment planning system
LDR: Low dose rate
GTV: Gross tumor volume
PTV: Planned target volume
PD: Prescription dose
D_90_: The minimum dose received by 90% of the PTV
V_100_: The percentage of the PTV receiving 100% of the prescription dose
V_150_: The percentage of the PTV receiving 150% of the prescription dose
D_mean_: The average radiation dose received by the tooth
D_max_: The highest radiation dose received by any part of the tooth
D_2cc_: The radiation dose received by the 2 cc of tooth with the highest exposure
DeI: Defect index
BT: Brachytherapy
SG: Surgery
ND: Not determined
OR: Odds ratios
NO: Teeth not reliably observed on radiograph
CI: Confidence interval
VIF: Variance inflation factor
