# Supplementary material for: The influence of brachytherapy on tooth development: a longitudinal study of pediatric head and neck tumor survivors
Source: BMC Oral Health. 2026 Mar 20;26:762. doi: 10.1186/s12903-026-07804-x (PMC13126827; doi:10.1186/s12903-026-07804-x)
Supplement: Supplementary file 1 — Supplementary Material 1 [file 12903_2026_7804_MOESM1_ESM.docx]

**Supporting Information**

**Table S1.**

Key characteristics of the surgery.

| No. | Treatment modality | Type of surgery | Extent of surgery |
| --- | --- | --- | --- |
| 2 | SG + BT | local excision  of the mass | inferior border of the right mandible,  right submandibular gland |
| 3 | SG + BT | segmental mandibulectomy | mandibular body distal to tooth #45,  ramus of the right mandible |
| 5 | SG + BT | local excision  of the mass | left parotid region |
| 6 | SG + BT | local excision  of the mass | left maxillary sinus |
| 7 | SG + BT | local excision  of the mass | mandibular body |

BT: brachytherapy; SG: surgery.

**Table S2.**

Key dosimetric characteristics of the brachytherapy.

| No. | Treatment modality | Radioactivity of seeds (mCi) | PD  (Gy) | D_90_  (Gy) | V_100_  (%) | V_150_  (%) |
| --- | --- | --- | --- | --- | --- | --- |
| 1 | BT | 0.50 | 110 | 122.1 | 91.5 | 51.9 |
| 2 | SG + BT | 0.60 | 110 | 122.2 | 91.7 | 53.3 |
| 3 | SG + BT | 0.50 | 120 | 114.4 | 84.1 | 33.1 |
| 4 | BT | 0.50 | 120 | 123.7 | 92.4 | 40.0 |
| 5 | SG + BT | 0.57 | 110 | 113.6 | 83.2 | 35.9 |
| 6 | SG + BT | 0.70 | 140 | 121.9 | 91.6 | 53.2 |
| 7 | SG + BT | 0.48 | 110 | 113.4 | 80.2 | 29.6 |

BT: brachytherapy; SG: surgery; PD: prescription dose; D_90_: the minimum dose received by 90% of the PTV; V_100_: the percentage of the PTV receiving 100% of the prescription dose; V_150_: the percentage of the PTV receiving 150% of the prescription dose.

**Table S3.**

Distribution of tooth development stage at start of brachytherapy.

| Dental position | Tooth developmental stage at start of brachytherapy | | | | | | | | | | |
| --- | --- | --- | --- | --- | --- | --- | --- | --- | --- | --- | --- |
|  | NO | 0 | 1 | 2 | 3 | 4 | 5 | 6 | 7 | 8 | 9 |
| Central incisors |  |  |  | 4 | 6 | 8 | 6 | 2 |  |  |  |
| Lateral incisors | 5 |  | 2 |  | 10 | 7 | 2 |  | 1 |  |  |
| Canines | 1 | 1 | 2 |  | 16 | 4 | 3 |  |  |  |  |
| First premolars | 4 |  |  | 8 | 8 | 5 | 2 |  |  |  |  |
| Second premolars | 4 | 4 |  | 12 |  | 4 | 4 |  |  |  |  |
| First molars |  |  |  | 4 | 5 | 10 | 2 |  | 4 |  |  |
| Second molars | 7 | 3 | 8 | 1 | 2 | 1 | 3 |  |  |  |  |
| Third molars | 21 | 2 | 2 |  |  |  |  |  |  |  |  |

NO: teeth not reliably observed on radiograph.

**Table S4.**

Grading of dental adverse events in cases based on CTCAE v6.0.

| No. | Dental caries | Tooth development disorder |
| --- | --- | --- |
| 1 | NO | Grade 2 |
| 2 | NO | Grade 3 |
| 3 | NO | Grade 3 |
| 4 | Grade 1 | Grade 3 |
| 5 | NO | NO |
| 6 | NO | Grade 2 |
| 7 | NO | Grade 2 |

Dental caries: grade 1, one or more dental caries, not involving the root; grade 2, dental caries involving the root; grade 3, dental caries resulting in pulpitis or periapical abscess or resulting in tooth loss; tooth development disorder: grade 1, Asymptomatic, hypoplasia of tooth or enamel; grade 2, impairment correctable with oral surgery; grade 3, maldevelopment with impairment not surgically correctable, limiting self-care ADL or severe impact on age-appropriate normal daily activity (pediatric); NO, adverse events not reliably observed.

**Figure S1.**

Figure S1. Receiver operating characteristic (ROC) curve for the multivariate logistic regression model of factors associated with dental abnormalities.
